# Seasonal Dynamics of Physiological, Oxidative and Metabolic Responses in Non-lactating Nili-Ravi Buffaloes Under Hot and Humid Climate

**DOI:** 10.3389/fvets.2020.00622

**Published:** 2020-09-08

**Authors:** Mengwei Li, Faiz-ul Hassan, Yanxia Guo, Zhenhua Tang, Xin Liang, Fang Xie, Lijuan Peng, Chengjian Yang

**Affiliations:** ^1^Key Laboratory of Buffalo Genetics, Breeding and Reproduction Technology, Ministry of Agriculture and Guangxi Buffalo Research Institute, Chinese Academy of Agricultural Sciences, Nanning, China; ^2^Institute of Animal and Dairy Sciences, University of Agriculture, Faisalabad, Pakistan

**Keywords:** temperature humidity index, heat stress, physiological response, oxidative stress, serum metabolites, buffalo

## Abstract

Hot and humid weather exposes animals to high temperature and relative humidity that ultimately reduce their ability to disperse body heat. To avoid serious consequences of heat stress, it is imperative to understand animal physiological responses and biochemical changes during a state of altered body homeostasis across different seasons of the year. This study evaluated seasonal dynamics of physiological, oxidative, and metabolic responses of Nili-Ravi buffaloes to hot and humid climate. Twenty non-lactating multiparous buffaloes were enrolled for this 1-year study. Meteorological data were recorded twice daily to calculate temperature humidity index (THI). Physiological parameters including rectal temperature (RT), body surface temperature (BST), and respiratory rate (RR) were measured weekly. Blood samples were collected once in each season (spring, summer, autumn, and winter) to analyze biochemical and antioxidant parameters. We also measured activities of liver enzymes including alanine aminotransferase (ALT) and aspartate aminotransferase (AST). The results revealed a significantly higher THI value (82) during summer which resulted in a significant increase in RR and BST as compared to winter. Higher oxidative stress was observed in summer owing to significantly higher malondialdehyde (MDA) content and lower levels of serum antioxidant enzymes (GPx, SOD, and CAT) as compared to other seasons. Moreover, serum cortisol was also significantly higher while adrenocorticotropic hormone (ACTH), Triiodothyronine (T_3_), insulin, and growth hormone contents were significantly lower in summer. Contrarily, plasma thyroxin (T_4_) level was higher in summer. THI showed a positive correlation with physiological responses but a negative correlation with antioxidant parameters. Our study provides practical insights on the adaptive physiology of buffaloes and has several implications regarding the alleviation of heat stress in buffaloes to enhance the efficiency of production and reproduction under tropical climate. Our study suggests the use of appropriate cooling strategies to effectively manage the non-lactating buffaloes to avoid performance losses and animal welfare issues in summer season.

## Introduction

Climate change imposes adverse effects on animal physiology, leading to an overall decrease in efficiency of production and reproduction. Moreover, it also raises animal health and welfare concerns, sometimes with serious consequences. Heat stress is one of the major climatic effects faced by animals, especially in tropical and sub-tropical countries. Heat stress in the tropics is generally associated with animal welfare issues and significant economic losses resulting from reduced performance but increased morbidity and mortality of livestock (1). The impact of heat stress is expected to become worse in the recent climate change scenario as it will increase the potential intensity of hot and humid conditions in the future, leading to an increased frequency of heat stress episodes (2). At higher ambient temperatures (above 30°C), excessive heat load hampers the ability of animals to dissipate their body heat. Heat stress occurs usually when animals are unable to maintain the balance between heat produced/stored and heat dissipated (3, 4). To manage this excessive heat load, animals attempt to reduce metabolic heat production while increasing heat dissipation to maintain euthermia. This is accompanied by a series of physiological, metabolic, and behavioral manifestations to thrive and mitigate adverse effects of heat stress. The first line of response to excessive heat load mainly includes accelerated respiratory rate (RR), increased water intake but reduced feed intake (5). Physiological parameters like body surface temperature (BST), rectal temperature (RT), respiratory rate, and pulse rate (PR) are the quick, ultimate responses of animals to climatic stress and eventually the level of discomfort or comfort of animals in a given environment (6).

Heat stress exposes dairy animals, especially buffaloes, to an excessive load of reactive oxygen species (ROS), leading to severe oxidative stress that subsequently reduces metabolic activity and immune response (7). Antioxidant system of animals fails to scavenge a large number of free radicals in time due to reduced activities of antioxidant enzymes (CAT, SOD, and GPx) under chronic heat stress (8). Therefore, levels of oxidative and antioxidant enzymes in ruminants can be used as markers to assess the degree of oxidative stress (9). Exposure to high ambient temperature also adversely affects the activity of the thyroid gland owing to its high sensitivity to environmental heat variations, subsequently leading to reduced levels of T_3_ and T_4_ (10). This decline in thyroid activity is coupled with a decrease in growth hormone to reduce metabolic activity and body heat increment. Moreover, stimulation of the hypothalamus pituitary–adrenal axis leads to the secretion of adrenocorticotropic hormone (ACTH) from the pituitary gland and then triggers the synthesis and secretion of glucocorticoids like cortisol (11). Hence, serum cortisol has been used extensively as a biomarker of heat stress in cattle (12, 13) and buffalo (14, 15).

These physiological manifestations in response to heat stress, significantly affect biological rhythm (diurnal and seasonal) in dairy animals. Compared to Zebu cattle, these effects are more pronounced in buffaloes owing to their dark skin color and lack of sweat glands. Heat stress triggers many physiological changes in the body of buffaloes, including a decrease in feed intake, shift in hormonal and metabolic secretions but an increase in BST, RT, RR, and oxidative stress (16, 17). Thermal comfort is generally measured by temperature-humidity index (THI) and has been applied to different livestock species, to understand the physiological stages of thermo-neutral and heat stress zones. It is imperative to better understand the changes in physiological and metabolic rhythms in buffaloes during different seasons, to devise strategies to mitigate the adverse effects of heat stress so that to avoid economic losses and animal welfare issues. Moreover, the association of THI with various physiological and biochemical parameters in buffaloes is a precondition to predict environmental effects during different seasons of the year. It is pertinent to mention that tropical climate of Southern China is quite different from other South Asian countries due to quite higher humidity which substantially increases average THI. Moreover, to best of our knowledge, no study is available regarding the seasonal variations in the oxidative physiology of non-lactating buffaloes under hot and humid climate of south China. Therefore, this study was planned to explore variations in biological rhythms of physiological, metabolic, hormonal, and oxidative responses of non-lactating Nili-Ravi buffaloes to tropical climatic conditions in Southern China.

## Materials and Methods

### Animal Welfare Statement

All procedures used in this experiment were approved by the Ethics Committee of the Guangxi Buffalo Research Institute, Chinese Academy of Agriculture Sciences China (Approval No. BRI2017006). All steps of animal rearing, recording of physiological parameters and blood sample collection were conducted strictly according to the guidelines of the committee. We confirm that we followed the rules of the Declaration of Helsinki for recording biological rhythms in buffaloes during this experiment.

### Geographical Location and Climate

This study was conducted at the Guangxi Buffalo Research Institute (22°53′22.59″ N; 108°21′51.19″ E; 122 m above sea level) located in Nanning, China. The overall climate of this area is characterized as hot and humid as it is located in the subtropical monsoon climate zone. This area receives sufficient sunshine with abundant rainfall (average 1,304 mm) with longer summer and short winter seasons. We used an online dust monitoring system (Shenzhen Greenford Environmental Technology Co., Ltd.) to record weather data in real-time, mainly including air temperature (AT, °C) and relative humidity (RH, %), with an interval at 30 min and at an installation height of buffalo's body.

**Figure 1:**
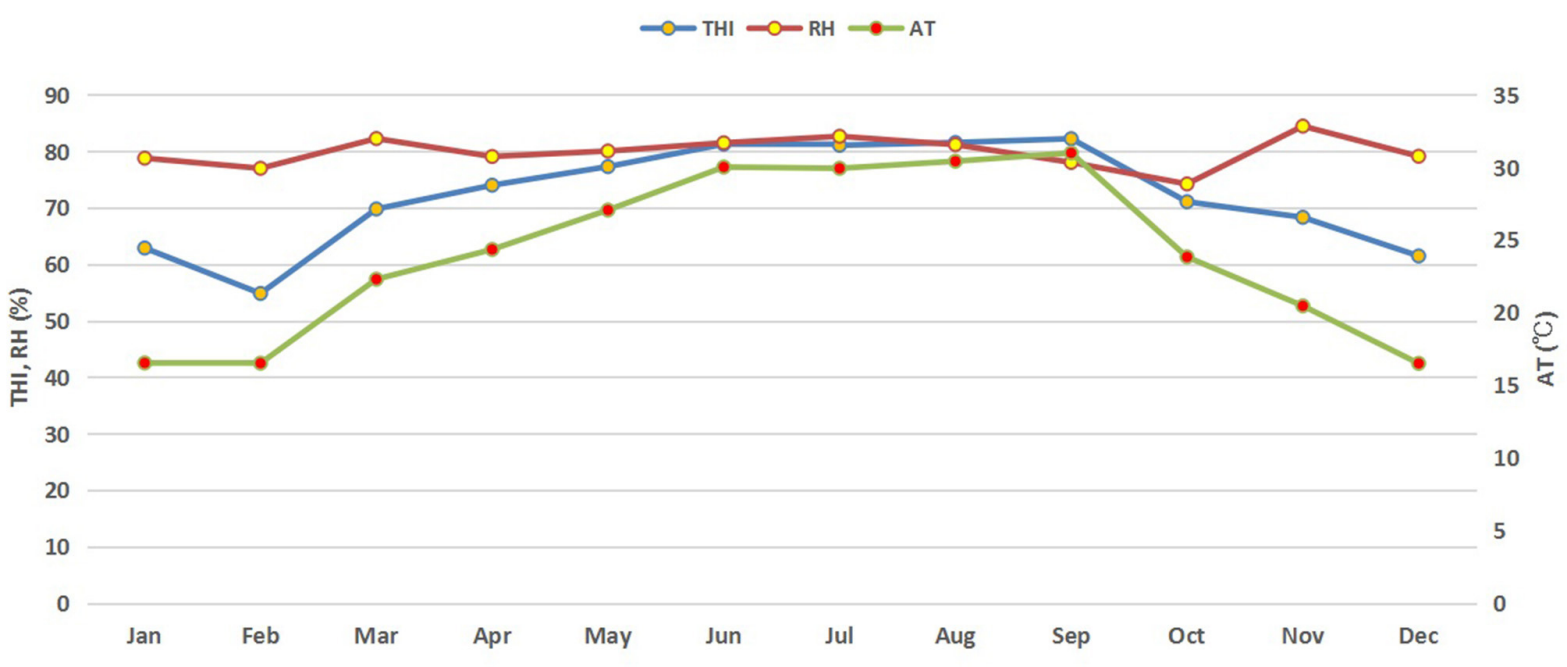
Monthly trends of THI, AT, and RH during the study period. THI, temperature humidity index; RH, relative humidity (%); AT, air temperature (°C).

Environmental variables were recorded twice daily in the morning (at 8:00 A.M.) and in the afternoon (at 2:30 P.M.) and used to depict monthly meteorological variations during the whole study period (Figure 1). Average daily temperature and relative humidity were recorded during the study period from April 2017 to March 2018 (for 1 year) and Temperature Humidity Index (THI) was calculated by using the following formula (18):

$$\begin{array}{l} \text{THI}=\text{AT}+0.36\text{ DPT}+\;41.5 \end{array}$$

Where, AT is the air temperature and DPT the dew point temperature of the buffalo shed. Four seasons of the year were defined as; spring (March–May), summer (June–August), autumn (September–November), and winter (December–February).

### Selection and Management of Buffaloes

Twenty non-lactating Nili-Ravi buffaloes were randomly selected for this experiment. All buffaloes were multiparous (3–5 lactations) with an average body weight of 550 ± 50 Kg. Buffaloes were housed in an open uncovered area with a stocking density of 15 m^2^/head throughout the study period. Free access to water was provided to all buffaloes throughout the day. Mist spraying was performed on buffaloes whenever the temperature exceeded 28°C throughout the year. All buffaloes were fed a total mix ration (TMR) consisting of grass (*Pennisetum purpureum schum*), corn silage and concentrate mixture (corn, soybean meal, and wheat bran). The same quantity of TMR was fed daily to each animal to meet its dietary requirement as per the general routine of the buffalo farm. The formulation and chemical composition of total mix ration are presented in Table 1.

**Table 1:** Feed composition and nutrient levels of the diet (air-dry basis,%).

**Ingredient**	**Content (%)**
Grass (*Pennisetum purpureum schum*)	29.6
Silage corn	48.2
Corn	16
Soybean meal	2
Wheat bran	1
Lime stone	0.5
CaHPO_4_	0.5
NaHCO_3_	0.7
NaCl	0.5
Premix^a^	1
Total	100
**Nutrient level^b^**	
CP	8.96
NDF	46.34
ADF	25.68
Ash	5.65

a *The additive premix provided the following elements per Kg of diets: Vitamin A 550,000 IU, Vitamin E 3,000 IU, Vitamin D3 150,000 IU, Fe (as ferrous sulfate) 4.0 g, Cu (as copper sulfate) 1.3 g, Mn (as manganese sulfate) 3.0 g, Zn (as zinc sulfate) 6.0 g, and Co (as cobalt sulfate) 80 mg*.

b *Measured values*.

### Recording of Physiological Parameters and Blood Sampling

Physiological parameters of buffaloes were recorded weekly as follows:

#### Body Surface Temperature (BST)

Average BST was recorded each Tuesday at 8:00 A.M. and 2:30 P.M. using an animal infrared thermometer from three different body sites, including forehead, left chest, and abdomen. Three respective values were averaged to get weekly BST for each buffalo.

#### Rectal Temperature (RT)

At the same time, RT was also recorded by inserting a veterinary rectal thermometer in the rectum of buffalo for 2 min.

#### Respiratory Rate (RR)

Weekly RR was recorded as times/min by observing and counting thoracic movements using a stopwatch and a counter for 2 min.

### Blood Sampling and Determination of Biochemical Parameters

Blood samples (10 mL) from each buffalo were collected from jugular vein before morning feeding once during each season viz: spring (April 17th), summer (July 22nd), autumn (October 18th), and winter (January 28th). These samples were used to determine serum hormones and antioxidant enzymes as follows:

#### Serum Hormones

Blood samples were put on ice after the collection and immediately transferred to the laboratory for serum isolation. Blood samples were centrifuged at 3,000 rpm for 15 min, and serum was harvested according to standard methods (19). Isolated serum was stored at −20°C until further analysis. Serum hormones including Adrenocorticotropic hormone (ACTH), Insulin, Cortisol, Triiodothyronine (T_3_), Thyroxine (T_4_), and growth hormone (GH) were analyzed using commercial ELISA kits according to manufacturer's instructions (CUSABIO BIOTECH CO., Wuhan, China). The coefficient of variation (inter and intra-assay) of these kits was <10% for each assay.

#### Serum Antioxidant Enzymes

Concentrations of serum antioxidant enzymes including total antioxidant capacity (TAC), malondialdehyde (MDA), superoxide dismutase (SOD), Glutathione peroxidase (GPx), and catalase (CAT) were determined through spectrophotometer using the Nanjing Built-in Kits (www.njjcbio.com) according to manufacturer's instructions. Average values for each enzyme in each season for all buffaloes are expressed as Mean ± s.e. The coefficient of variations (inter and intra-assay) of these kits was in the same range (<10%).

#### Plasma Biochemical Parameters

Plasma biochemical parameters including total protein (TP), albumin (ALB), globulin (GLB), blood urea nitrogen, and glucose were determined using respective commercially available kits according to manufacturer's instructions. Activities of liver enzymes ALT and AST were also determined using standard kits according to manufacturer's instructions.

### Statistical Analysis

Data were analyzed using the general linear model by analysis of variance in SAS software version 9.2 (SAS Institute Inc., Cary, NC, USA). Pearson correlation coefficients between THI and different physiological and biochemical parameters were calculated using the CORR command in SAS.

## Results

### Monthly Trend of Meteorological Data

**Figure 2:**
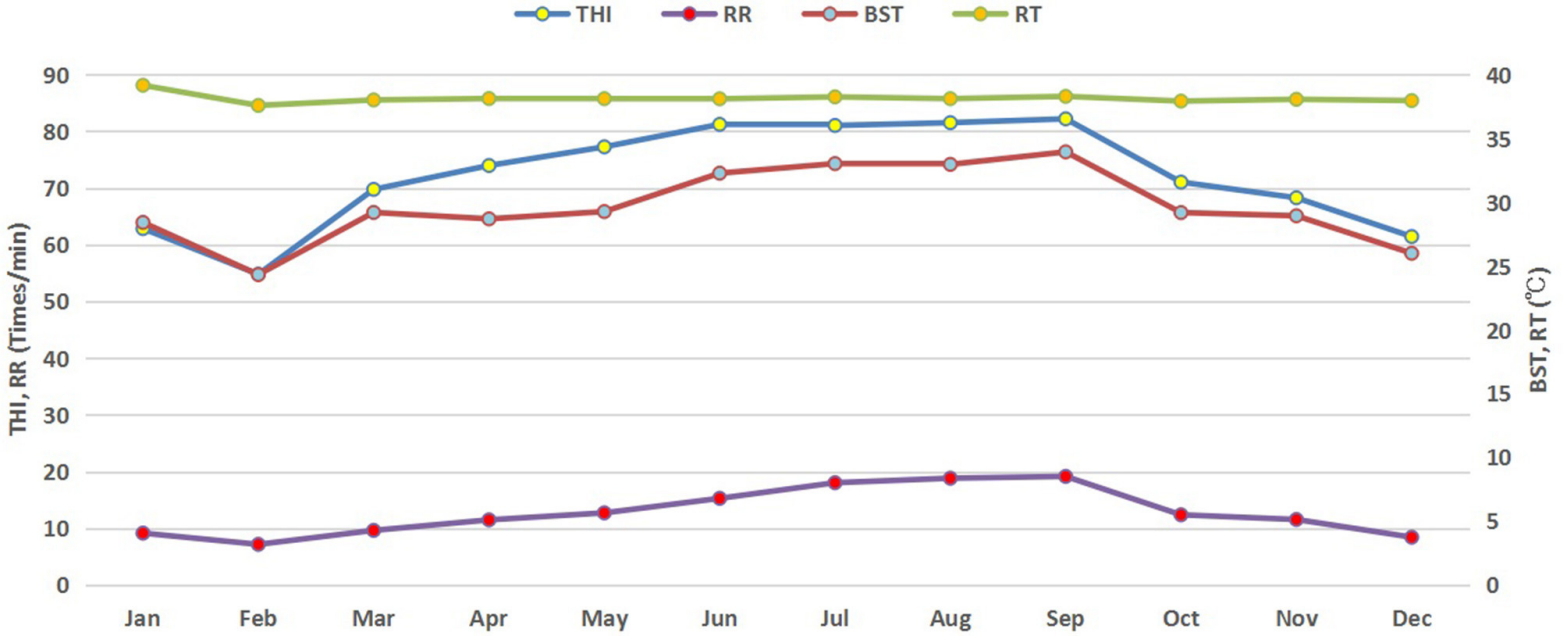
Monthly trends of physiological parameters of buffaloes in response to THI. THI, temperature humidity index; RT, rectal temperature (°C); RR, respiratory rate (times/minute); BST, body surface temperature (°C).

Monthly variations in THI, ambient temperature and relative humidity of the buffalo house are presented in Figure 1. Higher average THI values (above 80) were observed in summer (June–September) but started to decrease in autumn and winter. It started increasing again in spring. Similar trends in RR and BST of lactating buffaloes were also observed (Figure 2).

### Effect of THI on Physiological Parameters in Different Seasons

The average values of THI and physiological parameters of non-lactating buffaloes in different seasons are provided in Table 2. The THI indices in spring, summer, and autumn were significantly higher than that in winter (*p* < 0.05), with the highest value in summer, followed by autumn and spring. Moreover, RR, BST, and RT were all the highest in summer, while RT did not change across different seasons (*p* < 0.05).

**Table 2:** THI and physiological indices of non-lactating buffaloes in different seasons.

**Parameters**	**Spring**	**Summer**	**Autumn**	**Winter**
Temperature humidity index (THI)	74.49 ± 4.38^a^	82.00 ± 0.53^a^	75.51 ± 6.86^a^	60.25 ± 4.73^b^
Respiratory rate (times/min)	13.27 ± 1.72^ab^	17.32 ± 1.09^a^	15.16 ± 2.82^ab^	10.61 ± 3.14^b^
Body surface temperature (°C)	29.53 ± 0.35^b^	33.17 ± 0.52^a^	31.37 ± 2.50^ab^	25.96 ± 1.25^c^
Rectal temperature (°C)	38.11 ± 0.11	38.26 ± 0.04	38.18 ± 0.16	38.04 ± 0.06

*Different superscripts in same row indicate significant difference at p < 0.05*.

### Effect of Season on Serum Antioxidant Enzymes

**Figure 3:**
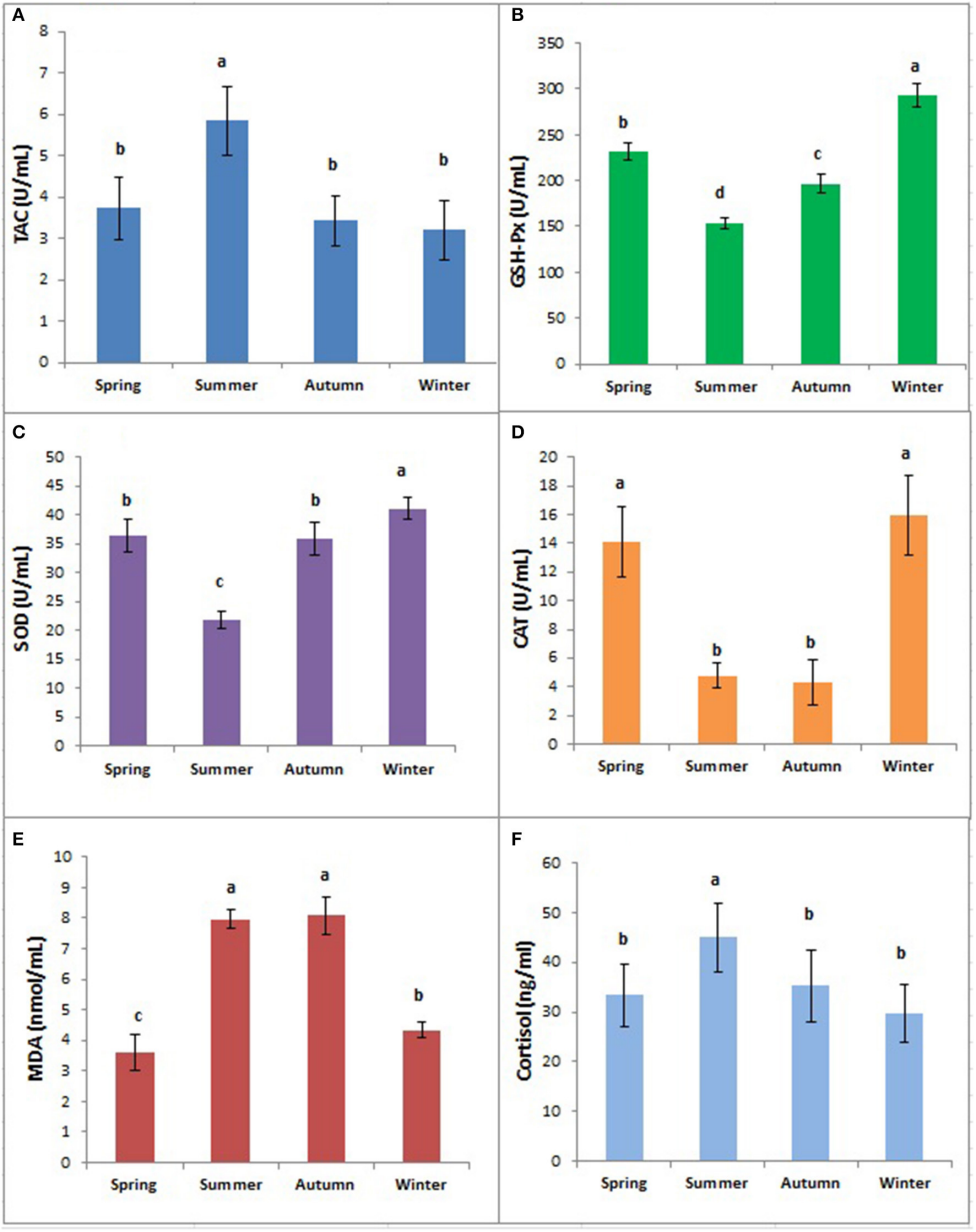
Concentrations of TAC **(A)**, GPx **(B)**, SOD **(C)**, CAT **(D)**, MDA **(E)**, and Cortisol **(F)** in buffaloes in different seasons. Bars marked with different letters differ significantly (*p* < 0.05). TAC, total antioxidant capacity; GPx, glutathione peroxidase; SOD, superoxide dismutase; CAT, catalase; MDA, malondialdehyde.

MDA concentrations were significantly (*p* < 0.05) higher in summer and autumn than in winter and spring (Figure 3). TAC was significantly higher in summer than in other seasons (*p* < 0.05) while the differences between spring, autumn, and winter were not significant (*p* > 0.05). However, serum concentrations of GSH, SOD, and CAT were significantly lower in summer but significantly higher in winter compared to other seasons (*p* < 0.05) as presented in Figure 3.

### Effect of Season on Serum Hormones

Significantly higher ACTH was observed in winter than other seasons (*p* < 0.05) (Table 3). However, cortisol was significantly higher in summer than in other seasons (*p* < 0.05). The insulin concentration in summer was significantly lower than other seasons (*p* < 0.05). Furthermore, GH and T_3_ concentrations were significantly lower in summer and autumn as compared to spring and winter (*p* < 0.05). Contrarily, T_4_ concentration was significantly higher in summer and autumn than in spring and winter (*p* < 0.05).

**Table 3:** Seasonal changes in serum hormone concentrations of non-lactating buffaloes.

**Items**	**Spring**	**Summer**	**Autumn**	**Winter**
Corticotropin (ACTH) (pg/ml)	84.31 ± 9.52^b^	55.44 ± 11.29^c^	84.94 ± 16.42^b^	101.21 ± 14.81^a^
Insulin (nIU/ml)	11.50 ± 1.64^a^	8.71 ± 2.24^c^	11.11 ± 2.18^ab^	9.89 ± 2.04^bc^
Triiodothyronine (T_3_) (ng/ml)	1.57 ± 0.61^a^	0.93 ± 0.34^b^	1.05 ± 0.26^b^	1.33 ± 0.21^a^
Thyroxine (T_4_) (ng/ml)	45.43 ± 9.23^b^	53.11 ± 13.70^a^	55.96 ± 8.03^a^	42.12 ± 4.42^b^
Growth hormone (ng/ml)	13.28 ± 2.21^a^	9.52 ± 3.84^b^	9.93 ± 3.25^b^	13.21 ± 4.39^a^

*Different superscripts in same row indicate significant difference at p < 0.05*.

### Effect of Season on Serum Biochemical Parameters

Total protein contents were higher in summer followed by spring, autumn, and winter while albumin was significantly higher (*p* < 0.05) in winter than other seasons (Table 4). Globulin was significantly higher in summer than spring and autumn followed by winter at the end (*p* < 0.05). Alanine aminotransferase (ALT) enzyme activity was significantly higher in summer than in other seasons (*p* < 0.05). Aspartate aminotransferase (AST) enzyme activity was significantly higher in spring than in other seasons (*p* < 0.05). Urea nitrogen was significantly lower in autumn than in other seasons (*p* < 0.05). Glucose was significantly higher in winter and autumn followed by summer and then winter at the end (*p* < 0.05) (Table 4).

**Table 4:** Blood biochemical parameters of non-lactating buffaloes during different seasons.

**Parameter**	**Spring**	**Summer**	**Autumn**	**Winter**
Total protein (g/L)	79.46 ± 5.78^ab^	82.36 ± 2.87^a^	77.18 ± 2.91^bc^	74.15 ± 5.59^c^
Albumin (g/L)	35.06 ± 3.10^b^	34.49 ± 1.79^b^	34.03 ± 2.06^b^	38.63 ± 2.41^a^
Globulin (g/L)	44.40 ± 5.23^b^	47.87 ± 2.65^a^	43.14 ± 2.45^b^	34.08 ± 3.83^c^
Alanine aminotransferase (U/L)	36.36 ± 9.34^b^	35.41 ± 6.43^b^	44.18 ± 3.40^a^	36.92 ± 5.58^b^
Aspartate aminotransferase (U/L)	125.18 ± 17.23^a^	108.91 ± 13.08^b^	112.81 ± 10.92^b^	107.14 ± 9.70^b^
Blood urea nitrogen (mmol/L)	6.32 ± 1.56^a^	7.00 ± 1.01^a^	5.16 ± 0.83^b^	6.44 ± 0.71^a^
Glucose (mmol/L)	2.57 ± 0.27^c^	3.46 ± 0.18^b^	3.73 ± 0.20^a^	3.92 ± 0.35^a^

*Different superscripts in same row indicate significant difference at p < 0.05*.

### Correlation of THI With Physiological and Biochemical Parameters

At significant levels (*p* < 0.05 or 0.01), Pearson correlation analysis showed positive associations of THI with all three physiological parameters of the non-lactating buffaloes, while RT, BST, and RR also positively correlated between each other. Negative correlations (*p* < 0.05) of THI with ACTH and cortisol were also observed. Both cortisol and T_4_ were positively correlated each other first and then with all three physiological parameters. Cortisol was negatively correlated with ACTH while T_3_ showed a positive correlation with GH (Table 5).

**Table 5:** Correlations of THI with physiological and biochemical parameters of buffaloes.

**Item**	**THI**	**BST**	**RT**	**RR**	**ACTH**	**Cortisol**	**Insulin**	**T_3_**	**T_4_**	**GH**
THI	1									
BST	0.938^**^	1								
RT	0.849^**^	0.846^**^	1							
RR	0.917^**^	0.951^**^	0.767^**^	1						
ACTH	−0.629^*^	−0.405	−0.118		1					
Cortisol	−0.624^*^	0.865^**^	0.833^**^	0.905^**^	−0.783^**^	1				
Insulin	−0.005	0.207	0.515	0.19	0.185	0.073	1			
T_3_	−0.264	−0.145	0.183	−0.112	0.14	0.164	0.554	1		
T_4_	0.412	0.687^*^	0.702^*^	0.784^**^	−0.499	0.814^**^	0.2	0.293	1	
GH	−0.236	−0.023	0.316	−0.057	0.09	0.261	0.505	0.848^**^	0.326	1

* *Shows significant correlation at p < 0.05*,

** *shows highly significant correlation at p < 0.01*.

*THI, temperature humidity index; BST, body surface temperature; RT, rectal temperature; RR, respiratory rate; ACTH, adrenocorticotropic hormone; T3, triiodothyronine; T_4_, Thyroxine; GH, growth hormone*.

### Correlation of THI With Serum Antioxidant Enzymes

THI showed highly negative correlations (*p* < 0.05 or 0.01) with GPx, SOD, and CAT but a positive correlation (*P* < 0.05) with TAC in the non-lactating buffaloes (Table 6). TAC content showed negative correlations (*p* < 0.05 or 0.01) with GPx and SOD and MDA content was negatively associated with GPx and CAT (*p* < 0.01). However, positive collections (*P* < 0.05 or 0.01) were observed between GPx, SOD, and CAT (Table 6).

**Table 6:** Correlation of THI with serum antioxidant parameters of non-lactating buffaloes.

**Items**	**THI**	**TAC**	**MDA**	**GPx**	**SOD**	**CAT**
THI	1					
TAC	0.640^*^	1				
MDA	0.54	0.522	1			
GSH	−0.837^**^	−0.608^*^	−0.736^**^	1		
SOD	−0.729^**^	−0.709^**^	−0.554	0.890^**^	1	
CAT	−0.596^*^	−0.353	−0.877^**^	0.871^**^	0.677^*^	1

* *Indicate significant correlation at p < 0.05*,

** *indicate highly significant correlation at p < 0.01*.

*TAC, total antioxidant capacity; MDA, malondialdehyde, SOD, superoxide dismutase, GPx, glutathione peroxidase; CAT, catalase*.

## Discussion

Climate is one of the major factors that cause oxidative stress and challenge the defense system of an animal, subsequently leading to many physiological manifestations. These physiological changes are the immediate responses of an animal to climatic stress and eventually reflect the level of discomfort or comfort of an animal in a given environment (6). The most obvious physiological effects of heat stress on animals include pronounced changes in their heart and respiratory rates. This metabolic and physiological load induced by heat stress also significantly affects their diurnal and seasonal rhythms (1).

### Effect of THI on Physiological Parameters

Our study revealed significant increases in BST and RR of non-lactating buffaloes in summer when THI value went up to 82. Increased RR and pulse rate (PR) reflected the response of animals, as an adaptive strategy, to lower down their body temperatures through evaporative cooling. Studies have reported higher RR and RT but lower PR in buffaloes exposed to high temperatures in a climatic chamber (3) and under pasture grazing system (20). Similarly, dramatic increases in RR, RT, and PR have been observed in cattle under heat stress conditions (21). However, average RT showed no significant difference among different seasons in the present study. Similar findings have been reported in swamp buffaloes at an ambient temperature of 30°C (22, 23). Exposure to high ambient temperatures (>30°C) has shown to stimulate a remarkable increase in RR and subsequent the initiation of panting without any change in RT in buffaloes (22). In our study, despite the high THI in summer, no severe response was found in physiological parameters of the non-lactating buffaloes, including RT as an index of heat storage in the body. This shows that thermo-tolerant ability of the non-lactating buffaloes along with management practices like mist spraying facilitated the mitigation of adverse effects of heat stress under tropical weather.

### Serum Antioxidant Indices of the Non-lactating Buffaloes in Different Seasons

Concentrations of antioxidant enzymes and heat shock proteins are one of the most important stress markers reflecting animals' comfort and climatic adaptability (24). We observed a significant increase in serum TAC in the non-lactating buffaloes in summer, indicating their ultimate response of antioxidant enzyme system to oxidative stress to remove excessive ROS. However, activities of CAT, SOD, and GPx were decreased significantly in summer, owing to the inability of antioxidant defense system to combat excessive ROS load (8). That's why such serum antioxidant enzymes have been used as physiological biomarkers to reveal the extent of oxidative stress in animals (9). Similarly, we observed significantly high concentrations of MDA in summer and autumn, revealing a higher degree of oxidative stress as it is a major end product of lipid peroxidation in cells to damage cell membranes. Similar findings regarding high MDA levels in summer have been reported earlier in Murrah buffaloes (25). In our study, a combination of oxidant (MDA) and antioxidant (TAC, GHS-Px, SOD, and CAT) enzymes revealed the high level of oxidative stress in non-lactating buffaloes during summer. These data indicate the need to exploit some cooling practices more effective than mist spraying to completely eliminate adverse effects of heat stress in buffaloes, especially during summer when THI value exceeds 80.

### Effect of THI on Hormonal and Biochemical Parameters of the Non-lactating Buffaloes

Exposure of an animal to high ambient temperature adversely affects the activity of its thyroid gland owing its great sensitivity to body temperature and metabolic heat increment (26). In our study, GH concentrations were decreased significantly in summer and autumn, revealing the most obvious sign of heat stress in the non-lactating buffaloes. Reduced plasma GH in summer was in agreement with earlier studies on buffaloes (27, 28). Plasma T_3_ levels were also decreased in summer and autumn which is in agreement with earlier findings regarding the depression of T_3_ in buffaloes during the summer stress period (29). Decreases in plasma T_3_ and T_4_ during heat stress were considered as an adaptive strategy to reduce basal metabolic rate and then net body heat production (30, 31). Contrary to our findings, no difference in plasma T_3_ and T_4_ in Nili-Ravi buffaloes was observed between hot humid and hot dry seasons (32). Fluctuations in T_3_ and T_4_ concentrations in response to heat stress are not abrupt as it takes time to change them and subsequently it requires several days to stabilize them at a new steady state (6).

In contrast to T_3_, plasma T_4_ was significantly higher in summer and autumn than in spring and winter. Similar findings have been reported earlier as a significantly higher level of T_4_ was observed in Khuzestan buffalo bulls during the summer season as compared to winter (33). Moreover, it was reported that the concentration of T_3_, TSH, and free T_4_ index did not differ between summer and winter seasons in same study. Additionally, no difference between T_3_ and T_4_ levels and their ratio has been observed in different seasons (summer, monsoon, and winter) in Murrah buffalo bulls (34). Similarly, no difference in T_3_ uptake (%) was observed in the summer and winter season in Friesian calves (35). But heat stress significantly reduced the T_4_ level in summer as compared to winter season while T_3_ uptake (%) showed no difference between summer and winter seasons as reported in buffaloes and Friesian cows (36). But in the same study, animals that were treated with water spray and a diaphoretic showed a 32.4% increase in T_4_ levels (42% in free T_4_ index) making its concentration significantly higher than observed in the summer season (35). Interestingly, T_3_ concentrations (% T_3_ uptake) showed a comparatively lower response to this treatment as only a 11% increase in its concentration was observed. Many other studies have also reported that use of evaporative cooling strategies (such as mist spraying or water sprinkling over animal) can significantly alleviate the adverse effects of heat stress and mitigate the decline in T_4_ levels as observed in crossbred cows (37) and buffaloes (38). Keeping in view of above findings the possible reason for higher plasma T_4_ levels observed in the present study during summer and autumn is the use of mist spraying over buffaloes whenever the ambient temperature exceeds 28°C. The same trend was not observed for T_3_ levels because T_3_ is generally seen as the most metabolically active thyroid hormone and it has a shorter half-life in the blood than T_4_ (39). The provision of mist spraying or water sprinkling showed no effect on plasma T_3_ levels in summer season (40). Additionally, wallowing has also shown to decrease free T_3_ in swamp buffaloes (41). That's why we observed lower plasma T_3_ in the summer and autumn while higher T_4_ in Nili-Ravi buffaloes in the present study.

The liver enzymes ALT and AST are the most reliable markers of liver damage or necrosis. The ALT primarily exists in the liver, but AST exists in various tissues like heart, liver, kidney, and so on. In the present study, we observed similar ALT levels during spring, summer, and winter except for autumn during which higher ALT level was observed. However, a higher AST level was found in spring with no change in other seasons. These findings revealed no adverse effect of heat stress on liver health in buffaloes in the present study as values of ALT and AST were within normal physiological limits (42). Animal exposure to ambient temperature up to 30°C, have not shown any significant change in ALT and AST levels in crossbred cattle (43). These observations are in line with our findings as we observed no rise in levels of both liver enzymes during the summer season in buffaloes as the average ambient temperature did not exceed above 30°C in the present study. No changes in the concentration of AST have been observed in the Surti buffalo during summer and winter seasons. However, a significant increase in ALT content was observed in the summer (under hot dry and hot humid conditions when THI increased from 68 to 81) as compared to winter season (44). Similar findings regarding the increase in ALT in the summer season (hot periods of the year) have been reported in cattle (45, 46). Heat stress during metabolically demanding conditions including lactation, pregnancy, or growth can put adverse effects on liver physiology, which is not the case with dry buffaloes as no changes in liver enzymes were observed during the summer season in the present study. Both AST and gamma-glutamyl transferase (GGT) have shown excellent predictive value for liver damage in cattle but in case of buffalo GGT is reported to be a more reliable indicator of liver damage (47). As we did not measure GGT in this study which is a limitation of the present study and should be considered in future studies.

Significantly high plasma cortisol was observed in summer, suggesting a high level of oxidative stress. Stressful conditions like heat stress result in activation of hypothalamic–pituitary–adrenal axis (HPA) which results in increased secretion of hypothalamic peptides including corticotrophin releasing hormone (CRH) and arginine vasopressin (AVP) into pituitary portal circulation, where they stimulate ACTH secretion. This, in turn, triggers an increase in cortisol release from the adrenal gland to act on multiple targets to restore homeostasis (48). Increased plasma cortisol levels observed in summer in the present study, indicated enhanced activity of HPA to facilitate homeostasis and mitigate the adverse effects of heat stress. Similar findings regarding high cortisol contents in summer have been reported earlier in Egyptian buffaloes (1), Murrah buffaloes (17, 29), and Sahiwal cows (13). Moreover, elevated cortisol levels have been observed in crossbred buffaloes (Nili-Ravi × Murrah) as compared to purebred Mediterranean buffaloes (49).

Contrary to cortisol, we observed significantly higher level of ACTH in buffaloes during summer. Regulation of ACTH secretion within the HPA axis is a multifactorial process, with the hypothalamic peptides CRH and AVP being important physiological stimulators, and cortisol along with other adrenal glucocorticoids as a major inhibitory factor by feedback loops (50, 51). Inhibition of the ACTH response to CRH and AVP by cortisol depends on the length of exposure: fast (within seconds to minutes), intermediate (2–10 h) and slow (hours to days) feedback (50). Both fast and intermediate feedbacks inhibit ACTH release rather than synthesis, while the slow feedback appears to affect both ACTH release and synthesis. The higher plasma cortisol levels results in negative feedback to subsequently lower the plasma ACTH levels through mediating complex interaction with CRH and AVP (52). It has also been reported earlier that ACTH increases in response to the onset of stress and then decreases in spite of continuing heat stimulus, indicating a negative glucocorticoid feedback and a decrease in the glucocorticoid-binding transcortin (53). This is the possible reason for lower ACTH levels observed in buffaloes during summer when plasma cortisol was quite higher in the present study.

We observed significantly low plasma insulin level in summer, in agreement with an early report (54). However, contrary findings like increase (23) or no change in insulin concentration (55) have been reported in cows under heat stress. Such variations are mainly attributed to different metabolic status, nutrition, physiological stage and thermo-tolerance ability of the animals.

### Association of THI With Physiological and Biochemical Parameters

THI has been efficiently used to evaluate the level of heat stress in animals (56, 57). Our study showed positive associations of THI with physiological parameters (BST, RR, and RT) and serum TAC content. However, moderately negative correlations of THI with ACTH and cortisol were observed, a bit strange as early study has reported a positive association of THI with cortisol content (29). However, a negative correlation of THI with serum antioxidant enzymes was in agreement with earlier studies on dairy cows (57, 58) and buffaloes (29). The decrease in activity of antioxidant enzymes during summer revealed a reduced total antioxidant capacity to manage the excessive load of ROS which has been associated with reproductive acyclicity in buffaloes, owing to severe damage to follicular cells (59). Usually non-lactating buffaloes are neglected in terms of management of heat stress as compared to lactating animals, which subsequently affects their reproductive performance. In our study, we observed average THI above 80 in summer which was well-correlated with the physiological and oxidative responses of non-lactating buffaloes. Therefore, it is recommended that in summer, buffaloes should be allowed to swimming for some period, especially during the hot period of the day, to alleviate adverse effects of heat stress. Earlier studies have also suggested the use of cooling strategies during months from April to September to alleviate heat stress in dairy animals (60). To best of our knowledge, present study is the first report on the seasonal dynamics of physiological and metabolic responses of non-lactating Nili-Ravi buffaloes to oxidative stress under the tropical climate in South China. It provides practical insights on the adaptive physiology of buffaloes and has several implications regarding the alleviation of heat stress in buffaloes to enhance the efficiency of performance and avoid animal welfare issues under tropical climate. However, a relatively small sample size is a limitation of this study that needs to be accounted for in future studies.

## Conclusions

High THI in summer was associated with significant increases in physiological parameters, serum MDA and cortisol concentrations in non-lactating buffaloes. Moreover, THI showed a highly negative association with serum total antioxidant capacity as it significantly reduced serum contents of GSH, SOD, and CAT in summer. Plasma T_3_ and GH concentrations were also reduced in summer. Our study reveals that summer months (from June to September) are critical in buffalo management as THI value exceeds 80, so cooling practices are required to alleviate heat stress to avoid performance losses and animal welfare issues. However, further studies are required on a larger cohort of samples to corroborate the association of THI thresholds with physiological and biochemical parameters in the non-lactating buffaloes under tropical climatic conditions.

## Data Availability Statement

All datasets generated for this study are included in the article/supplementary material.

## Ethics Statement

The animal study was reviewed and approved by Ethics Committee of the Guangxi Buffalo Research Institute, Chinese Academy of Agriculture Sciences China (Approval No. BRI2017006). All animals studied were the property of the Guangxi Buffalo Research Institute.

## Author Contributions

ML, ZT, and CY: conceptualization. FH: data curation. ML: formal analysis. CY: funding acquisition. ML, ZT, FX, and LP: investigation. ML, YG, FX, and LP: methodology. XL: project administration. YG, ZT, XL, FX, LP, and CY: resources. FH: software and writing—original draft. CY: supervision and validation. FH and XL: visualization. ML and CY: writing—review and editing. All authors contributed to the article and approved the submitted version.

## Conflict of Interest

The authors declare that the research was conducted in the absence of any commercial or financial relationships that could be construed as a potential conflict of interest.
